# Population pharmacokinetics of ATR inhibitor berzosertib in phase I studies for different cancer types

**DOI:** 10.1007/s00280-020-04184-z

**Published:** 2020-11-04

**Authors:** Nadia Terranova, Mendel Jansen, Martin Falk, Bart S. Hendriks

**Affiliations:** 1 Translational Medicine, Merck Institute of Pharmacometrics, Lausanne, Switzerland, an affiliate of Merck KGaA, Darmstadt, Germany; 2 Clinical Pharmacology, QureQuest Ltd, London, UK; 3 Global Clinical Development, Merck KGaA, Darmstadt, Germany; 4 Translational Medicine, EMD Serono Research & Development Institute, Inc. (an affiliate of Merck KGaA, Darmstadt, Germany), Billerica, MA USA

**Keywords:** ATR inhibitor, Berzosertib, Population pharmacokinetics, DNA damage repair (DDR) inhibitors

## Abstract

**Purpose:**

Berzosertib (formerly M6620) is the first-in-class inhibitor of ataxia–telangiectasia and Rad3-related protein, a key component of the DNA damage response, and being developed in combination with chemotherapy for the treatment of patients with advanced cancers. The objectives of this analysis were to characterize the pharmacokinetics (PK) of berzosertib across multiple studies and parts, estimate inter-individual variability, and identify covariates that could explain such variability.

**Methods:**

A population PK analysis was performed using the combined dataset from two phase I clinical studies (NCT02157792, EudraCT 2013-005100-34) in patients with advanced cancers receiving an intravenous infusion of berzosertib alone or in combination with chemotherapy. The analysis included data from 240 patients across 11 dose levels (18–480 mg/m^2^). Plasma concentration data were modeled with a non-linear mixed-effect approach and clinical covariates were evaluated.

**Results:**

PK data were best described by a two-compartment linear model. For a typical patient, the estimated clearance (CL) and intercompartmental CL were 65 L/h and 295 L/h, respectively, with central and peripheral volumes estimated to be 118 L and 1030 L, respectively. Several intrinsic factors were found to influence berzosertib PK, but none were considered clinically meaningful due to a very limited effect. Model simulations indicated that concentrations of berzosertib exceeded p-Chk1 (proximal pharmacodynamic biomarker) IC_50_ at recommended phase II doses in combination with carboplatin, cisplatin, and gemcitabine.

**Conclusions:**

There was no evidence of a clinically significant PK interaction between berzosertib and evaluated chemo-combinations. The covariate analysis did not highlight any need for dosing adjustments in the population studied to date.

**Clinical Trial information:**

NCT02157792, EudraCT 2013-005100-34

**Electronic supplementary material:**

The online version of this article (10.1007/s00280-020-04184-z) contains supplementary material, which is available to authorized users.

## Introduction

Berzosertib (formerly M6620, VX-970, VE-822) is a selective and potent inhibitor of ataxia–telangiectasia and Rad3-related (ATR) for intravenous administration and is being developed in combination with chemotherapy for the treatment of patients with advanced cancers [1, 2]. The rationale for clinical development is based on the observation that deoxyribonucleic acid (DNA) damaging drugs and ionizing radiation are widely used as standard of care for the treatment of many solid tumors; however, for many patients, they provide only modest benefit due to highly proficient cellular processes that are able to detect and repair the damaged DNA. ATR is a serine/threonine kinase and critical regulator of the DNA damage response (DDR) involved in sensing DNA replication stress which may be caused by many factors including replication of unresolved DNA damage and oncogenic stress driving dysregulated replication [3].

ATR activation induces the phosphorylation of checkpoint kinase 1 (Chk1) and several other targets, leading to cell cycle arrest and multiple mechanisms that result in avoidance of double-strand DNA breaks, replication fork collapse, and mitotic catastrophe [4–8]. Inhibition of ATR results in unresolved replication stress leading to replication fork collapse and induction of potentially fatal double-strand breaks, eventually resulting in cell death. While normal cells can generally tolerate inhibition of ATR by activating compensatory DNA repair pathways, such pathways are frequently defective in cancer cells, rendering them highly dependent on ATR for survival. Increased reliance on ATR signaling also notably occurs in cancer cells following DNA damaging chemotherapy. Additional endogenous mechanisms may also increase reliance on ATR [9, 10], including defects or inhibition of other DNA damage pathways, overexpression of oncogenes, and/or utilization of the alternative lengthening of telomeres mechanism [9, 10]. Under these conditions, ATR inhibition may lead to lethal levels of DNA damage and rapid cell death.

Berzosertib is currently in phase I/II studies in combination with other anti-cancer treatments, specifically chemotherapeutic drugs. Multiple preclinical studies support the potential efficacy of berzosertib in combination with chemotherapy. Berzosertib dramatically enhanced the efficacy of cisplatin in multiple patient-derived non-small cell lung tumor xenografts [11]. In combination with gemcitabine, berzosertib acutely sensitized acute myeloid leukemia cells and increased survival in an orthotopic mouse model [12]. Berzosertib selectively sensitized pancreatic cancer cells but not normal cells to radiation and/or gemcitabine. It markedly prolonged growth delay of pancreatic cancer xenografts after radiation and gemcitabine-based chemoradiation without augmenting normal cell or tissue toxicity [13, 14]*.*

It is hypothesized that inhibition of ATR during its peak activation following DNA damage could maximize potential for benefit. In vitro and in vivo studies in combination with gemcitabine suggest that optimal administration of berzosertib is 12–24 h (h) after administration of the chemotherapy and corresponds to peak activation of p-Chk1, a pharmacodynamic (PD) biomarker for ATR activity [15, 16].

Preclinical pharmacokinetics (PK)/PD studies conducted with berzosertib in tumor-bearing mice evaluated inhibition of p-Chk1, a direct downstream substrate of ATR, in combination with various chemotherapies. Berzosertib mediated dose-dependent inhibition of p-Chk1 that correlated with efficacy and, further, did not show significant dependence on the chemotherapy partner it was combined with or dependence on the tumor model. From collective studies of berzosertib in combination with carboplatin, irinotecan, cisplatin, or gemcitabine in multiple preclinical tumor models, the total plasma concentration needed to reach 50% of maximal inhibition (IC_50_) of p-Chk1 in humans was estimated to be approximately 110 ng/mL (95% confidence interval (CI) 62–158 ng/mL) after adjustment for species differences in plasma protein binding [17].

In two phase I clinical studies, MS201923-0001 (“Study 001”, NCT02157792) [18] and VX13-970-002 (“Study 002”, EudraCT 2013-005100-34) [19], berzosertib tolerability and pharmacokinetics were evaluated as monotherapy and in combination with multiple chemotherapies, including gemcitabine, cisplatin, and carboplatin. For these combinations, berzosertib was administered approximately 1 day after administration of gemcitabine or platinum-based chemotherapy. PK data for berzosertib are available from these two studies. Berzosertib PK appeared linear over the dose range evaluated (18–480 mg/m^2^) with a terminal half-life of approximately 17 h. Elimination of berzosertib is believed to be via extensive metabolism based on in vitro data, mainly mediated by CYP3A4, which is supported by the limited renal excretion (5–6%, Study 001). Based on their respective absorption, distribution, metabolism, and excretion properties and evaluation of drug–drug interaction potential, no clinically significant PK interaction was expected with gemcitabine, cisplatin, or carboplatin.

The recommended phase II dose (RP2D) of berzosertib as determined by the safety and tolerability profiles obtained through dose escalation, differed depending on the chemotherapy to which berzosertib was added: 90 mg/m^2^ (days 2 and 9) following carboplatin area under the curve (AUC) 5 (day 1) [19], 140 mg/m^2^ (days 2 and 9) following 75 mg/m^2^ cisplatin (day 1) [18] and 210 mg/m^2^ (days 2 and 9) following 1000 mg/m^2^ gemcitabine (days 1 and 8) [20].

This study sought to provide an integrated characterization of berzosertib PK across multiple studies, alone and in combination with various chemotherapies. To this end, a population PK model was developed to describe the berzosertib concentration time-course following multiple infusions and assess the impact of intrinsic and extrinsic factors on the between-patient variability. The potential need for dose adjustments for specific sub-populations was included in the analysis and comparisons of human PK with preclinical measures of activity (at the RP2Ds for berzosertib) were further evaluated.

## Materials and methods

### Clinical trials and analysis set

Longitudinal berzosertib plasma concentration and dosing data from the two phase I clinical trials, described above, were available for the analysis. The studies were performed in accordance with the Declaration of Helsinki; all participants provided written informed consent before blood samples were collected.

Study 001 (Online Resource Figure S1) was a phase I, open-label, first-in-human multiple ascending dose study to investigate the safety, tolerability, and PK of berzosertib alone and in combination with cytotoxic chemotherapy in patients with advanced solid tumors. The RP2D or maximum tolerated dose was determined in dose escalation parts A and B with further evaluation of safety and efficacy in expansion cohorts (parts C1–3). PK data were available from 170 patients who received twice-weekly 1-h intravenous infusions of berzosertib (18–210 mg/m^2^) during a 7–14-day monotherapy lead-in. This preceded combination with chemotherapies, administered on day 1, and berzosertib administered on days 2 and 9 every 21 days following (1) gemcitabine, or cisplatin and gemcitabine (54 patients, Part A); (2) cisplatin (29 patients, Part B); (3) gemcitabine in patients with non-small cell lung cancer (NSCLC; 36 patients, Part C1); (4) cisplatin in patients with triple-negative breast cancer (TNBC) basaloid subtype and BRCA wild type (37 patients, Part C2); cisplatin or carboplatin in patients with platinum-resistant small cell lung cancer (SCLC; 14 patients, Part C3). Berzosertib was also evaluated in combination with irinotecan in a subsequent study cohort (Part B2). In Study 001, Parts A and B, rich PK sampling (pre-dose, 0.5, 1 h (end of infusion), 1.5, 2, 4, 8, 24, 48, and 72 h after the start of infusion) was performed for most patients following berzosertib monotherapy in the lead-in. Similar rich PK sampling was conducted on cycle 1 days 2 through 5 after the first dose of berzosertib in combination with other agents in study parts A and B or during dose escalation. Additional sparse samples (pre-dose and 2 h after the end of infusion) were taken during cycle 1, day 9 and cycle 2, day 2. In the expansion cohorts Parts C1, C2, and C3, limited PK sampling was performed on cycle 1 day 2: pre-dose, 0.5, 1 h (end of infusion), 1.5, 2, 4, and (optionally) 8 h after the start of infusion. Similar sampling was performed on cycle 1, day 9 on a portion of the participants before being removed by amendment.

Study 002 (Online Resource Figure S1) was a phase I, open-label, multiple ascending dose study to investigate the safety, tolerability, and PK/PD profile of berzosertib as a single agent and in combination with carboplatin or carboplatin and paclitaxel in patients with advanced solid tumors. PK data were available from 70 patients who received 1-h intravenous infusions of berzosertib (1) as a single agent once weekly (11 patients, Part A1); (2) as a single agent twice weekly (6 patients, Part A2); (3) on days 2 and 9 every 21 days following carboplatin on day 1 (23 patients, Part B1); (4) on days 2 and 9 every 21 days following carboplatin and paclitaxel on day 1 (15 patients, Part B2); (5) on days 2 and 9 following carboplatin on day 1 every 21 days in patients with advanced solid tumors and lymphoma with defects in the DDR (15 patients, Part C). In Study 002, in all study parts, rich PK sampling (pre-dose, 0.5, 1 h (end of infusion), 1.5, 2, 4, 8, 24, and 48 h after start of infusion) was performed after the first dose of berzosertib. In Part A1, additional sparse samples (pre-dose, 0.5–2 h after the end of infusion) were collected on cycle 1, day 8 and day 15. For participants in Part A2, similar rich PK sampling was also conducted on cycle 1, day 8 and additional sparse samples were collected on cycle 1, day 4 (pre-dose, 0.5–2 h after the end of infusion), day 11 (pre-dose), and day 15 (pre-dose, 0.5–2 h after end of infusion) and cycle 2, day 1 (pre-dose, 0.5 h after start of infusion, 1 h (end of infusion) and 1 sample 0.5–2 h after the end of infusion). In Part B, additional sparse samples were collected on cycle 1, day 9 (pre-dose) and cycle 2, day 2 (pre-dose, 0.5 h after start of infusion, 1 h (end of infusion) and 1 sample 0.5–2 h after the end of infusion). In Part C, additional sparse samples were collected on cycle 1, day 9 (pre-dose) and cycle 2, day 2 (pre-dose, 0.5 h after the start of infusion, 1 h (end of infusion), and 0–2 h (1 sample) and 2–5 h (1 sample) after the end of infusion).

After exclusions due to missing data and observations below the lower limit of quantification (10 ng/ml), which represented a small percentage (< 10%), a total of 2557 PK concentration observations across 11 nominal dose levels (18–480 mg/m^2^) from 240 patients were available for modeling. Rich PK sampling up to three days after the first dose of berzosertib was conducted in all study parts, except for Part C1 of Study 001 where PK samples were taken up to 4 (or optionally 8) h post-beginning of infusion on days 2 and 9. Additional sparse samples were taken during cycles 1 and 2. Dose levels and patients included in this analysis are reported for each study part in Table 1.

**Table 1:** Overview of berzosertib PK analysis dataset by study and part

Study	Part	Design	Number of patients	Berzosertib dose levels
Study 001—dose escalation	Parts A and B	Berzosertib monotherapy lead-in only	2	72, 140 mg/m^2^ (day -14 and -7)
	Part A1	Berzosertib in combination with gemcitabine	45 (30 also in lead-in)	18, 36, 60, 72, 90, 140, 210 mg/m^2^ (day -14 or -7 for lead-in, then days 2 and 9 in a 21-day cycle)
	Part A2	Berzosertib in combination with gemcitabine and cisplatin	8	90, 120 mg/m^2^ (days 2 and 9 in a 21-day cycle)
	Part B	Berzosertib in combination with cisplatin	28 (2 also in lead-in)	90, 140, 210 mg/m^2^ (day -14 for lead-in, then days 2 and 9 in a 21-day cycle)
Study 001—expansion cohorts	Part C1 (NSCLC)	Berzosertib in combination with gemcitabine	36	210 mg/m^2^ (days 2 and 9 in a 21-day cycle)
	Part C2 (TNBC)	Berzosertib in combination with cisplatin or carboplatin	37	90, 140 mg/m^2^ (days 2 and 9 in a 21-day cycle)
	Part C3 (SCLC)	Berzosertib in combination with cisplatin or carboplatin	14	90, 140 mg/m^2^ (days 2 and 9 in a 21-day cycle)
Study 002—dose escalation	Part A1	Berzosertib monotherapy once weekly	11	60, 120, 240, 480 mg/m^2^ (on days 1, 8, and 15 in a 21-day cycle)
	Part A2	Berzosertib monotherapy twice weekly	6	240 mg/m^2^ (on days 1 and 4, 8 and 11, and 15 and 18 in a 21-day cycle)
	Part B1-1	Berzosertib in combination with carboplatin	23	90, 120, 240 mg/m^2^ (days 2 and 9 in a 21-day cycle)
	Part B1-2	Berzosertib in combination with carboplatin and paclitaxel	15	45, 90 mg/m^2^ (days 2 and 9 in a 21-day cycle)
Study 002—expansion cohort	Part C (DDR)	Berzosertib monotherapy followed by (upon progression) berzosertib in combination with carboplatin	15	240 mg/m^2^ twice weekly (days 1, 4, 8, 11, 15, 18 in a 21-day cycle) followed by 90 mg/m^2^ (days 2 and 9 in a 21-day cycle)

*DDR* patients with advanced solid tumors or lymphoma with DDR defects of interest, dose escalations were in participants with advanced solid tumors, *NSCLC* non-small cell lung cancer, *PK* pharmacokinetic, *SCLC* small cell lung cancer, *TNBC* triple-negative breast cancer, *lead-in* refers to a 14–7 day period of berzosertib monotherapy prior to cycle 1 day 1 and initiation of combination therapy

### Bioanalytical methods

Blood samples were collected in K_2_EDTA as an anticoagulant. Bioanalysis of Study 001 was conducted at MedPace (Cincinnati, OH, USA) and Study 002 at inVentiv Health (Princeton, NJ, USA) using similar methods in which berzosertib concentrations in plasma were quantified using validated liquid chromatography–tandem-mass spectrometry (LC–MS/MS) methods and an internal standard. The calibration range was 10–2500 ng/mL for both studies.

### Population PK analysis

#### Structural and statistical model development

Various structural PK models (one-, two-, and three-compartmental models with first-order linear elimination) were evaluated. To develop the statistical model, PK parameters were assumed to be log-normally distributed. The inter-individual variability (IIV) in model parameters was investigated by considering exponential error models for between-subject random effects. Additive, proportional, and combined error models were explored for residual unexplained variability.

#### Covariate analysis model development

Covariate relationships were assessed using the full covariate model approach, in which all covariates of interest were tested on the PK parameters, simultaneously [21]. Predefined covariates of interest included demographics (age, sex, body weight), laboratory values (serum albumin, platelets), renal impairment (defined according to the U.S. Food and Drug Administration guidance [22]), hepatic impairment (defined according to the National Cancer Institute Organ Dysfunction Working Group categorization [23]), Eastern Cooperative Oncology Group performance status (ECOG PS), tumor burden, tumor type. Race, ethnicity, aspartate transaminase, alanine transaminase, and serum bilirubin were graphically explored and included only if relevant trends with IIV estimates for the structural parameter of interest were seen. Although berzosertib is dosed by body surface area (BSA), bodyweight was selected as the weight-related covariate for easier interpretation in terms of the allometric coefficient. Only baseline covariates were assessed. Indeed, as there was no indication of any change of PK over time, no time-varying covariates were explored.

The 95% CIs for covariate relationships were calculated based on NONMEM standard errors (asymptotic 95% CIs). Forest plots were used to illustrate the influence of covariates on the model. Effects were not considered significant if the 95% CI for the parameter including the covariate effect overlapped the null value, and it was completely enclosed within the “no effect” range (defined as 80–120% of the point estimate of the covariate).

The effect of categorical covariates on a particular parameter P was linearly evaluated as follows:

$$P{ } = \theta_{1} \times \left( {1 + \theta_{k} \times {\text{CAT}}} \right),$$

where CAT is an indicator variable, which is equal to 0 for the most common category and to 1 for the *k*th category. Accordingly, *θ*_1_ is the typical estimate of the parameter for the most common category and *θ*_k_ is the fractional change in the typical parameter value for patients in the *k*th category.

The effects of continuous covariates were evaluated with a power function as follows:

$$P{ } = \theta_{1} \times \left( {\frac{{{\text{CON}}}}{{{\text{CON}}_{{{\text{median}}}} }}} \right){{\theta_{2} }}$$

where CON_median_ is the observed median CON, *θ*_1_ is the parameter estimate when CON equals CON_median_ and *θ*_2_ is the change in ln(P) per unit change in ln(CON) from ln(CON_median_).

The assessment of covariates effects in the forest plot used the less frequent categories as indicator variable CAT for categorical covariates and the extremes of observed distributions as indicator variable CON for continuous covariates.

#### Model-based simulations

Evaluations of different doses using the established model were conducted to investigate the relationship between berzosertib exposure and nonclinical pharmacology data. Based on the population PK model parameter estimates and associated uncertainty, model simulations were performed with the full covariate model for a virtual population of 5000 patients. Covariates were resampled jointly from distributions observed in the studies, thus, ensuring any correlations were kept in the simulated dataset. Resampled individual BSAs were used to determine the dose in mg for each virtual patient and at each considered dose level.

#### Modeling methodology and software

The population PK analysis was performed with a non-linear mixed-effects modeling approach using NONMEM (version 7.3.0; Icon Development Solutions, Hanover, MD, USA) [24] installed on LINUX (Novell SLES11 (64-bit) SP3) operating system, with CPU allocation controlled by a Univa Grid Engine (version 8.2), part of a validated GxP environment. The NONMEM runs on the servers were executed by Perl-speaks-NONMEM (PsN, version 4.4.8) [25, 26], which was also used to aid the development of the non-linear mixed-effect models as well as to perform model simulations using NONMEM. Pirana (version 2.9.2) [27] was used to organize the runs and produce their summary. The statistical software R (version 3.5.1) [28] as well as packages xpose4 (version 4.6.1) and xpose (version 0.4.3) [25] were used for the exploratory analysis and post-processing of NONMEM output, for example to assess the goodness-of-fit.

The NONMEM estimation method used was the first-order conditional with interaction [29]. The stability of NONMEM models was assessed on the basis of acceptable goodness-of-fit plots, number of significant digits ≥ 3 for all estimated parameters, successful covariance step, estimates of typical patient parameters not close to a boundary, and stability of the final solution to perturbations of the initial values. The selection of the structural model was based on objective function value (using a reduction of ≥ 10.83, corresponding to *α* = 0.001 with one degree of freedom), goodness-of-fit plots (e.g., relevant residuals against time randomly distributed around zero), and scientific plausibility of the parameter estimates. Visual predictive checks (VPCs) and prediction-corrected visual predictive checks (pcVPCs) were also generated at key model development decision points to evaluate the model predictive performance [30].

## Results

### Patient population

**Fig. 1:**
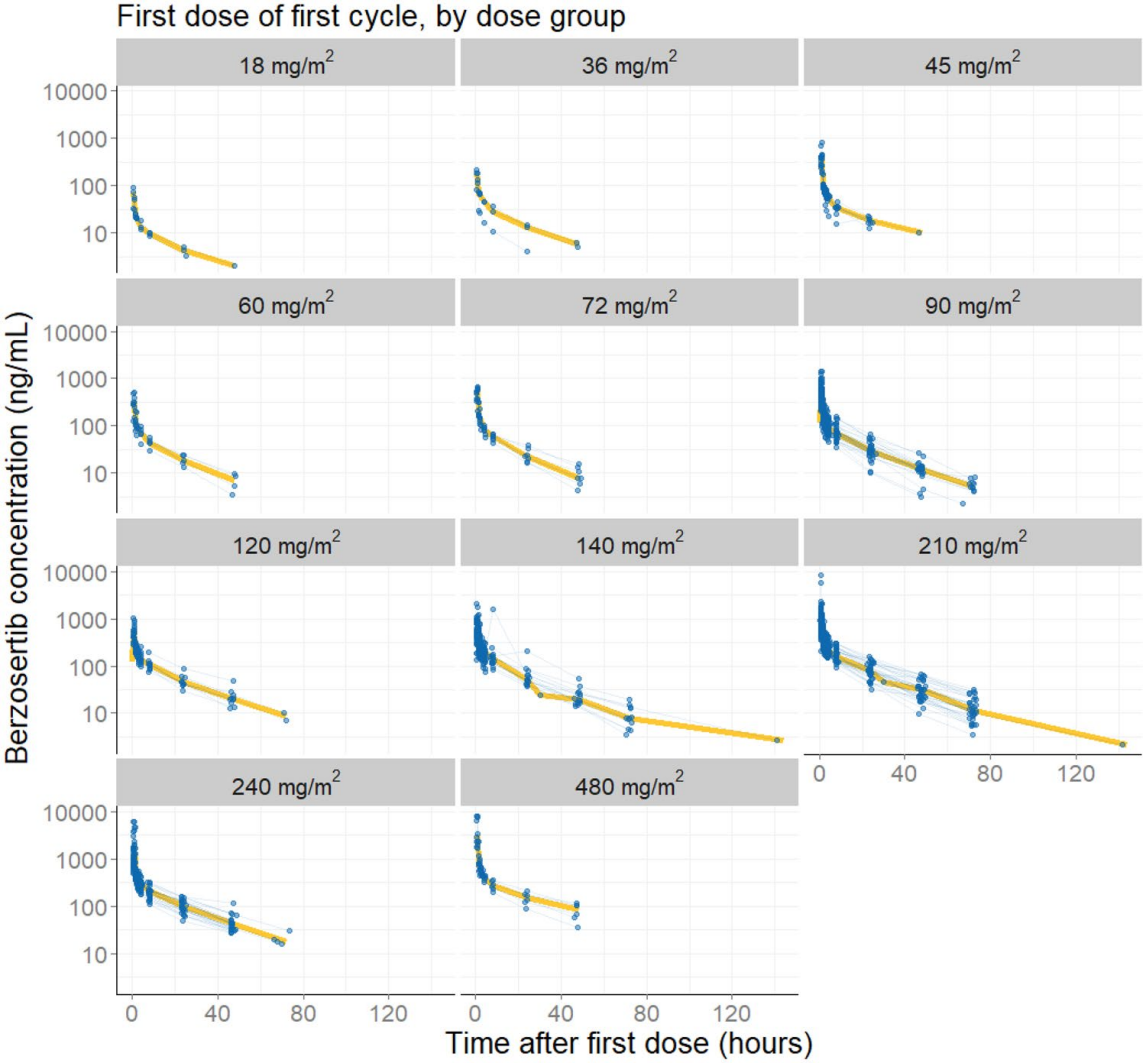
Berzosertib concentration profiles by dose levels administered in Study 001 and Study 002. Observed berzosertib concentrations are shown over time for each dose level. Blue points connected by lines are individual observations; the yellow line a smoothing curve

A summary of the baseline characteristics of all patients included in the analysis is provided in Table 2. Online Resource Table S1 provides summaries by study. PK profiles from a total of 240 patients were used in this analysis. Most patients were white (93.3%), female (60.4%), with median age of 60 years (range 26–79), and median body weight at baseline of 72.8 kg (range 46–150). About 68% of patients had an ECOG PS of 1 at baseline. After exclusions of remaining outliers based on the fit for the chosen model (conditional weighted residuals > 6), a total of 2546 concentration records (1417 and 1129 observations from Study 001 and Study 002, respectively) across 11 nominal dose levels (18–480 mg/m^2^) were available to provide the starting point for the base model development. Berzosertib concentration data over time across first dose levels are shown in Fig. 1. In Online Resource Figure S2, concentration profiles by dose level and combination agents are also shown.

**Table 2:** Demographics and baseline characteristics of combined participants in Studies 001 and 002

Variable	Value (*N* = 240)
Demographics	
Age [years]	60 {58.4} (26–79) [0]
Body weight [kg]	72.8 {75.4} (46–150) [0]
BSA [kg/m^2^]	1.82 {1.84} (1.4–2.59) [0]
Lean body mass [kg]	49.9 {52.3} (35.7–78.9) [1]
Height [cm]	168 {167} (99–191) [0]
Sex [*n* (%)]	
Male	95 (39.6%)
Female	145 (60.4%)
Race [*n* (%)]	
White	224 (93.3%)
Black	3 (1.25%)
Asian	5 (2.08%)
Other	6 (2.5%)
Missing	2 (0.833%)
Ethnicity [*n* (%)]	
Hispanic/latino	7 (2.92%)
Not hispanic/latino	226 (94.2%)
Missing	7 (2.92%)
Lab values	
Creatinine [µmol/L]	66.6 {69.8} (32.7–148) [0]
Creatinine clearance [mL/min]	95 {100} (50–150) [0]
Platelet count [10^9^/L]	274 {289} (93–816) [0]
White cell count [cells/µL]	7.2 {7.65} (1.37–25.2) [0]
Albumin [g/L]	38 {37.6} (23–49) [0]
Bilirubin [µmol/L]	7 {8.16} (0.5–21) [0]
ALT [U/L]	20 {26.9} (4–178) [0]
AST [U/L]	24 {31.1} (9–159) [0]
Renal impairment [*n* (%)]	
None	137 (57.1%)
Mild	88 (36.7%)
Moderate	15 (6.25%)
Severe	0 (0%)
Hepatic impairment [*n* (%)]	
None	166 (69.2%)
Mild	43 (17.9%)
Moderate	0 (0%)
Severe	1 (0.417%)
Missing	30 (12.5%)
Disease status	
Tumor burden [mm]	75 {84.1} (10–312) [0]
Tumor type [*n* (%)]	
NSCLC	48 (20.0%)
TNBC	33 (13.8%)
SCLC	16 (6.67%)
PrCa	7 (2.92%)
Breast	11 (4.58%)
H&N	1 (0.417%)
CRC	44 (18.3%)
Ovarian	9 (3.75%)
Mesothelioma	12 (5.0%)
Other	59 (24.6%)
ECOG PS [*n* (%)]	
0	65 (27.1%)
1	164 (68.3%)
2	5 (2.08%)
3	6 (2.5%)

Continuous covariates are reported as median {geometric mean} (range) [missing]. Categorical covariates are reported as number (percentage)

*ALT* alanine transaminase, *AST* aspartate transaminase, *BSA* body surface area, *CRC* colorectal cancer, *ECOG PS* Eastern Cooperative Oncology Group Performance Status, *H&N* head & neck, *NSCLC* non-small cell lung cancer, *PrCa* prostate cancer, *SCLC* small cell lung cancer, *TNBC* triple-negative breast cancer

### Base model

A two-compartmental linear model was found to best describe the concentration data of patients receiving berzosertib intravenously in the two considered studies. The best base model included IIV on clearance (CL), intercompartmental CL (Q), central (V1), and peripheral (V2) volume of distribution as a full variance–covariance matrix. The residual error was described by a combined additive and proportional error model. Parameter estimates of the base model are reported in Online Resource Table S2. VPCs showed good agreement between the original data and obtained model predictions (data not shown). The model re-run with the re-inclusion of previously identified outliers showed an impact only on the additive error which was estimated to significantly higher values.

### Full covariate model

Graphical explorations of variables against between-subject random-effects estimates for CL, V1, and V2 did not highlight relevant trends for variables (i.e., race, ethnicity, aspartate transaminase, alanine transaminase, and serum bilirubin) not included in the predefined set of covariates to be tested. Similarly, no obvious relationships were suggested from explorations of variables against IIV estimates for Q. Thus, only predefined demographics, laboratory values, disease-related measures, and baseline tumor information were included in the model CL, V1, and V2 with a full covariate approach. No covariates were tested on Q. In line with no expectation of PK drug–drug interaction, concentration profiles did not suggest any relationship with given combination agents (see Online Resource Figure S2). Thus, no covariates relating to co-administration of chemotherapy agents were included. Missing covariate values were set to the population median or to the most common category.

The full covariate model provided acceptable results as shown by the goodness of fits plots and pcVPC in Online Resource Figures S3 and S4. Although very limited data were available beyond 40 days, no bias of predictions related to time could be identified, thus suggesting a good description of PK data after multiple does of berzosertib without accounting for any changes of exposure over time. The population parameter estimates along with their uncertainty are reported in Table 3. Representative individual predicted concentration–time profiles overlaid with the observed concentrations are also shown from the two phase I studies in Online Resource Figure S5.

**Table 3:** Structural and random-effects parameter estimates for the full covariate model

Parameter	Estimate	RSE %^a^	Asymptotic 95% CI	Shrinkage (%)^b^
Clearance CL [L/h]	65	5.2	58–71	–
Central volume V1 [L]	118	12	91–150	–
Peripheral volume V2 [L]	1030	3.9	950–1100	–
Intercompartmental clearance Q [L/h]	295	3.5	270–320	–
IIV on CL [var]	0.066	6.5	0.049–0.083	8.7
cov (CL, V1)	0.060	12.0	0.031–0.089	–
cov (CL, Q)	0.054	16.0	0.021–0.087	–
cov (CL, V2)	0.041	7.3	0.029–0.052	–
IIV on V1 [var]	0.32	7.1	0.24–0.42	8.1
cov (V1, Q)	0.25	7.7	0.18–0.33	–
cov (V1, V2)	0.081	9.0	0.052–0.11	–
IIV on Q [var]	0.24	9.2	0.16–0.33	5.4
cov (Q, V2)	0.090	16.0	0.021–0.087	–
IIV on V2 [var]	0.047	7.9	0.033–0.062	6.8
Proportional residual error [sd]	0.22	4.6	0.2–0.24	–
Additive residual error [ng/mL]	1.73	19.0	1.1–2.4	–

*CI* confidence interval, *CL* clearance, *var* variance, *cov* covariance, *sd* standard deviation, *IIV* inter-individual variability, *Q* intercompartmental clearance, *RSE* relative standard error, *V1* central volume of distribution, *V2* peripheral volume of distribution

^a^Obtained by NONMEM covariance step. The relative standard errors for individual variability parameters are reported on the approximate standard deviation scale (standard error/variance estimate)/2

^b^The epsilon shrinkage was estimated to 10%

**Fig. 2:**
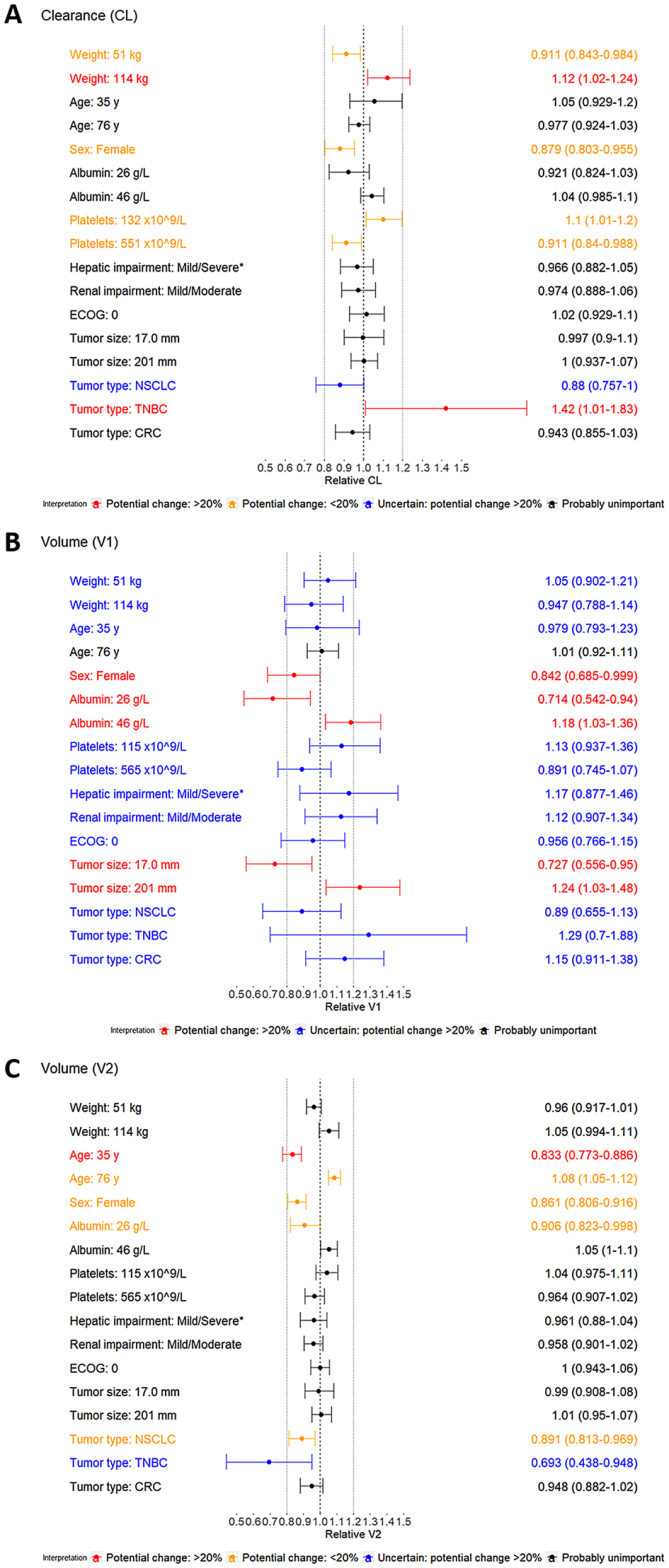
Forest plots of covariate effects on CL and V1 for the full covariate model. *CL* clearance, *CRC* colorectal cancer, *ECOG* Eastern Cooperative Oncology Group, *NSCLC* non-small cell lung cancer, *TNBC* triple-negative breast cancer, *V1* central volume of distribution, *V2* peripheral volume of distribution. *One single patient presented severe status thus precluding the estimation of the effect of this hepatic impairment category

The influence of several intrinsic factors on berzosertib PK could be identified using a full covariate model approach, but these factors were not considered clinically meaningful, because their overall effects were limited. Forest plots showing the size of the effects (including uncertainty) of the covariates on the different parameters in the model are presented in Fig. 2. For continuous covariates, the effects are shown for the covariate extremes defined as the 2.5th and 97.5th percentile of the observed distributions. For categorical covariates, the effects are shown for the less frequent categories which, given the limited number of patients across them, combined in one category mild and severe status (the latter represented by a single patient) for hepatic impairment, and mild and moderate status for renal impairment. ECOG PS of 0 was tested versus the most frequent PS of 1. No categories were tested for status greater than 1 which were combined to the most frequent category due to the very limited occurrences.

Results highlighted the effects of tumor type and bodyweight on CL. In particular, patients with TNBC had 42% higher CL than female patients with other tumor types. Bodyweight was also an influential covariate with a median increase of 12% and a decrease of 9% in CL, at high and low extremes of body weight, respectively. For V1, a median increase of 24% at high extremes of tumor size at baseline was suggested. The V1 in female patients was predicted to be 15.8% lower than in male patients. A median decrease of 28.6% for V1 was observed for the low extreme of albumin, while 18% increase was suggested for the high extreme. A median decrease of 16.7% for V2 was observed at the low extreme of age. Smaller but still potentially relevant effects were observed for sex and platelet count for CL, and sex, albumin, and tumor type for V2. High uncertainty was associated with the estimation of effects of tumor type on CL and V2, as well as all remaining covariates on V1. Overall, the addition of covariates could explain only 3–6% of the variability of model parameters, and it did not provide any improvement in terms of relative standard errors which were already low in the base model. As shown in Online Resource Figure S4, the pcVPC of the full covariate model was acceptable, thus confirming model suitability for simulations. Similar results, also in terms of covariates effects, were obtained when re-running the full covariate model with BSA included in place of body weight (data not shown). When additional data will be available from berzosertib development, the full covariate model or more complex approaches (e.g., full random-effects model) may be assessed to obtain a final model including only relevant covariates.

### Simulation of different dosing levels

The relationship between berzosertib exposure and efficacy in the context of combination with chemotherapy, including the timing of ATR inhibition relative to chemotherapy in humans, as well as the extent and duration of target inhibition required for maximal efficacy, is not fully understood. For this reason, we performed simulations of representative berzosertib dose levels in humans (administered intravenously over 1 h), corresponding to the different RP2Ds determined in combination with different chemotherapies. The p-Chk1 IC_50_, determined from preclinical studies to be approximately 110 ng/mL (adjusted for interspecies differences in plasma protein binding), provides a point of reference to compare concentrations with estimated compound potency [17].

**Fig. 3:**
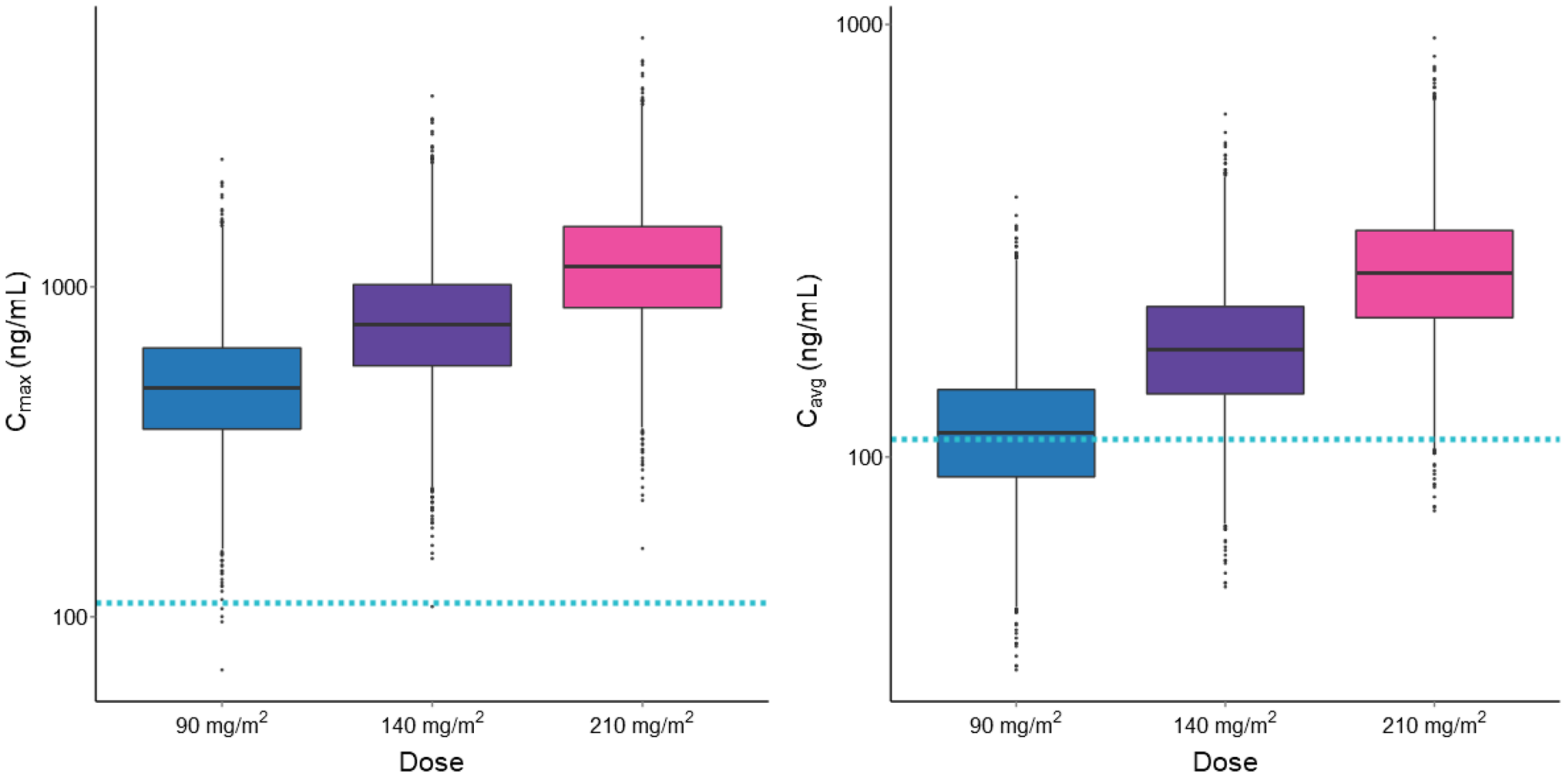
Distribution of simulated exposure metrics following berzosertib recommended dose for different combination regimens. *C*_*max*_ maximum concentration, *C*_*avg*_ average concentration. Distributions of simulated C_max_ (left) and *C*_avg_ (right) following berzosertib single dose of 90 mg/m^2^ (recommended dose for the combination with carboplatin), 140 mg/m^2^ (recommended dose for the combination with cisplatin), and 210 mg/m^2^ (recommended dose for the combination with gemcitabine). Boxes represent interquartile range; horizontal lines are first quartiles, medians, third quartiles; lower whiskers extend from each quartile to the extreme non-outlier values in the dataset; points are outliers. The blue horizontal dashed line represents the p-Chk1 IC50

PK profiles were simulated for virtual patients treated with berzosertib single dose of 90 mg/m^2^, 140 mg/m^2^, and 210 mg/m^2^, corresponding to the RP2D for berzosertib in combination with carboplatin, cisplatin, and gemcitabine, respectively. Average (C_avg_) and maximum concentration (C_max_) after a single dose of berzosertib were derived and their distributions are shown in Fig. 3. A strong inhibition of p-Chk1 is predicted with these berzosertib dose levels as suggested by the mean C_max_ over IC_50_ ratios equal to 4.8 for 90 mg/m^2^, 7.5 for 140 mg/m^2^, and to 11.2 for 210 mg/m^2^. Maximum concentrations of berzosertib exceeded p-Chk1 IC_50_ across all dose levels with mean time above it of 2.5 h (95% CI 1–8 h) for 90 mg/m^2^, 8 h (95% CI 2–20 h) for 140 mg/m^2^, and 16 h (95% CI 8–28 h) for 210 mg/m^2^. Furthermore, average concentrations consistently exceeded p-Chk1 IC_50_ in 54%, 91%, and 99% of the virtual subjects treated with 90 mg/m^2^, 140 mg/m^2^, and for 210 mg/m^2^, respectively.

## Discussion

Berzosertib is a selective inhibitor of ATR, currently in clinical development as part of combination therapies for various indications. The ability to combine this class of agents with standard of care chemotherapy is of potential high interest. Understanding sources of variability is of key importance in enabling broad clinical development and building future combinations. This work provides an integrated analysis of berzosertib population PK across multiple chemotherapy combinations.

Population PK model development confirmed dose and time linear PK, moderate-to-high clearance, and extensive tissue distribution of berzosertib following intravenous administration in patients with advanced solid tumors. Graphical exploration of the data indicated no clear deviation from linearity of berzosertib PK across all doses evaluated without any apparent impact of co-administered agents. Furthermore, evaluation of distributions of post hoc PK parameter estimates did not suggest differences in berzosertib PK across dose levels and combination therapies. The base structural PK model was a two-compartment linear model and described the data well. Goodness-of-fit and model assessment criteria suggested that the full covariate model was consistent with the observed data without any apparent systematic bias. A full covariate model approach for covariate modeling was used and provided acceptable results. For a typical patient, the CL and intercompartmental CL (Q) were estimated to be 65 L/h and 295 L/h, respectively, with V1 and V2 of 118 L and 1030 L, respectively.

The influence of intrinsic and extrinsic patient factors on PK is important to guide further development and optimize dosing, as drug safety and effectiveness can vary with PK. The effect of several intrinsic factors on the PK of berzosertib was identified, but was determined not to be clinically meaningful. The most influential covariates on CL were tumor type with 42% higher CL in patients with TNBC and bodyweight with a median increase of 12% at the high extreme. Tumor size at baseline, sex, and albumin presented the strongest association with V1, while age was the most influential covariate on V2. Potential effects were also observed for sex and platelet count for CL, and for sex, albumin, and tumor type for V2. Although the effects of TNBC and NSCLC tumor types were estimated on CL and V2, respectively, an association between tumor type and PK is not anticipated. Overall, berzosertib exhibits moderate variability in PK, given its intravenous administration. The addition of covariates could explain only 3–6% of the variability in the model parameters. Part of the unexplained variability may be attributable to variability across patients in CYP3A4 activity, the hypothesized primary route of berzosertib elimination, and is consistent with previous studies in cancer patients [31, 32].

Simulations for berzosertib RP2Ds in combination with cytotoxic drugs were performed to evaluate the relationship between PK and a nonclinical measure of potency using the full covariate model. Maximal concentrations were consistently above the p-Chk1 IC_50_ predicted from preclinical models with significantly increased duration above it with increasing dose level. These data suggest potential for a PD effect of berzosertib at these dose levels. Importantly, these highlight that more robust and more durable target inhibition is expected with increasing dose of berzosertib. Therefore, maximizing berzosertib exposure in combination with chemotherapy would be expected to maximize potential for efficacy. Consequently, chemotherapy (and other) combinations that can tolerably support higher berzosertib doses (gemcitabine and/or topotecan [33]) may have an increased probability of success.

Preclinical studies elucidated the importance of timing relative to administration of chemotherapy with the apparent optimum of ATR inhibitor administration at approximate peak onset of p-Chk1 response to chemotherapy-induced DNA damage. The predicted PK of berzosertib relative to p-Chk1 IC_50_ implies that inhibition of ATR is expected to be transient with the current schedules used in combination with chemotherapy. It may further highlight the importance of timing of berzosertib to coincide with potential peak chemotherapy-induced p-Chk1 activation for optimal activity. Consequently, the kinetics and duration of the DNA damage response elicited by various chemotherapies in patients is hypothesized to be of key importance for combination with ATR inhibitors. The importance of the timing and duration of ATR inhibition in combination with chemotherapy in patients is incompletely understood and merits further research.

In addition, a range in patient tumor sensitivity to ATR inhibition is also expected and the ability to identify patient subgroups may be critical for therapeutic success. Further preclinical and translational studies investigating the PK/PD/efficacy and safety relationships for inhibition of ATR in combination with chemotherapy under different doses and schedules are warranted. However, the exposure–response relationship for clinical safety would have to be characterized for each combination to inform the optimal dosing.

In summary, this analysis describes the population PK of berzosertib given alone or with various combination agents in patients with different advanced solid tumors. The present data and analysis suggest that dose modifications are not likely to be needed based on the covariates evaluated. However, weight-related measures did not appear to be strong covariates based on currently available data, and thus, their clinical relevance should be further assessed when more data will be available from berzosertib development. Similarly, as not all covariate effects could be precisely estimated with the limited amount of data available, the presence of significant relationships may emerge in future assessment. Analysis in subgroups beyond those studied (e.g., patients with moderate or severe hepatic or renal impairment) may be necessary. The population PK model developed herein provides a key starting point for subsequent investigations, including exposure–response analyses for ongoing and future trials.

## Electronic supplementary material

Below is the link to the electronic supplementary material.

Supplementary file1 (DOCX 1039 kb)

## Data Availability

Any requests for data by qualified scientific and medical researchers for legitimate research purposes will be subject to the Merck KGaA, Darmstadt, Germany Data Sharing Policy. All requests should be submitted in writing to the Merck KGaA, Darmstadt, Germany data sharing portal (https://www.merckgroup.com/en/research/our-approach-to-research-and-development/healthcare/clinical-trials/commitment-responsible-data-sharing.html). When Merck KGaA, Darmstadt, Germany has a co-research, co-development, or co-marketing or co-promotion agreement, or when the product has been out-licensed, the responsibility for disclosure might be dependent on the agreement between parties. Under these circumstances, Merck KGaA, Darmstadt, Germany will endeavor to gain agreement to share data in response to requests.
