# Supplementary material for: Population pharmacokinetics of ATR inhibitor berzosertib in phase I studies for different cancer types
Source: Cancer Chemother Pharmacol. 2020 Nov 4;87(2):185–96. doi: 10.1007/s00280-020-04184-z (PMC7870753; doi:10.1007/s00280-020-04184-z)
Supplement: Supplementary file 1 — Supplementary file1 (DOCX 1039 kb) [file 280_2020_4184_MOESM1_ESM.docx]

# **Online resources**

**Fig. S1** Summary of study designs


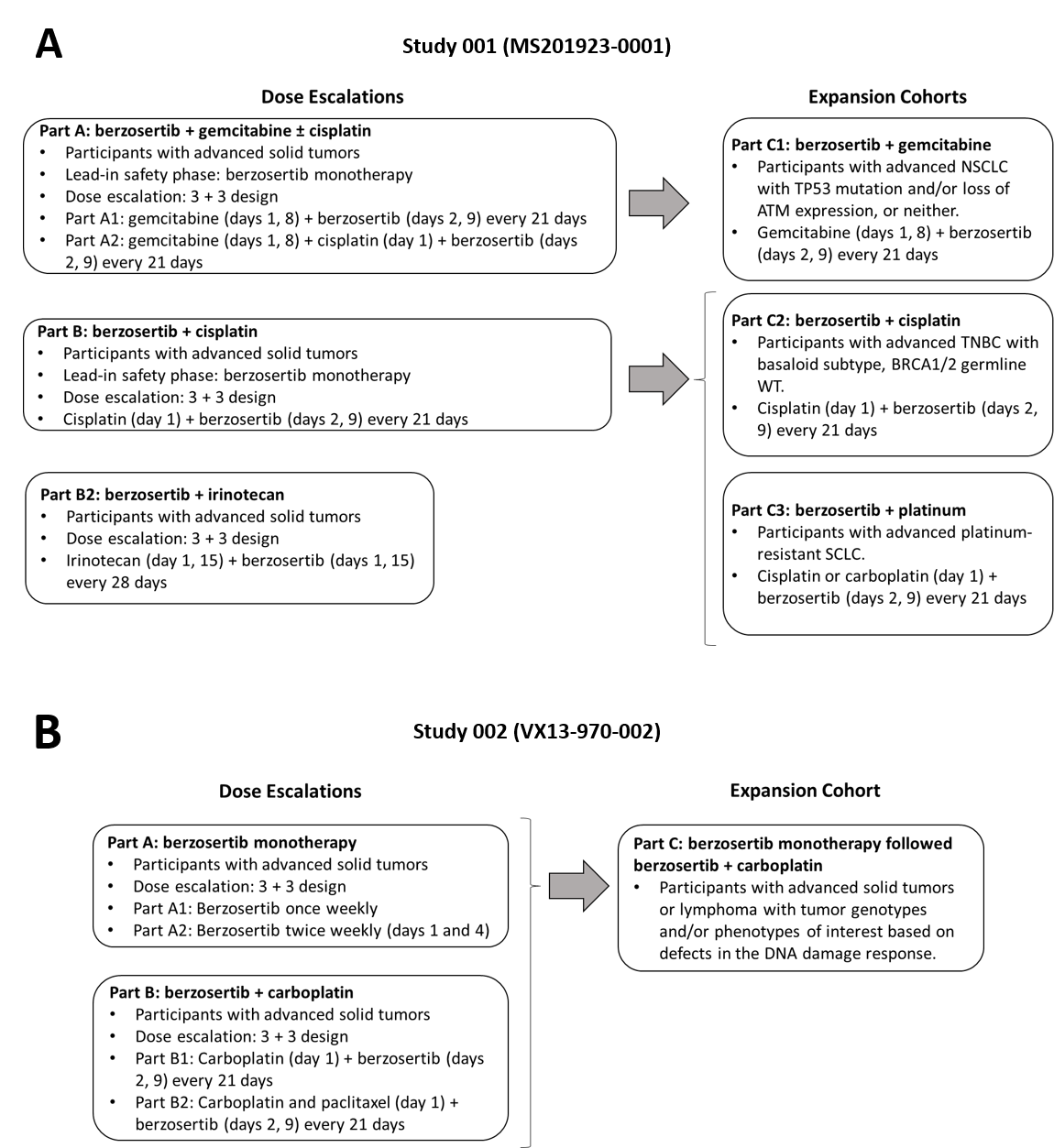


Designs of Study 001 and Study 002 are illustrated in panels A and B, respectively. The population PK analysis included data from all Berzosertib study parts, except Part B2 as those data were not available at the time of the analysis.

**Fig. S2** Berzosertib concentration profiles after the first dose by dose levels and combination arm in Study 001 and Study 002
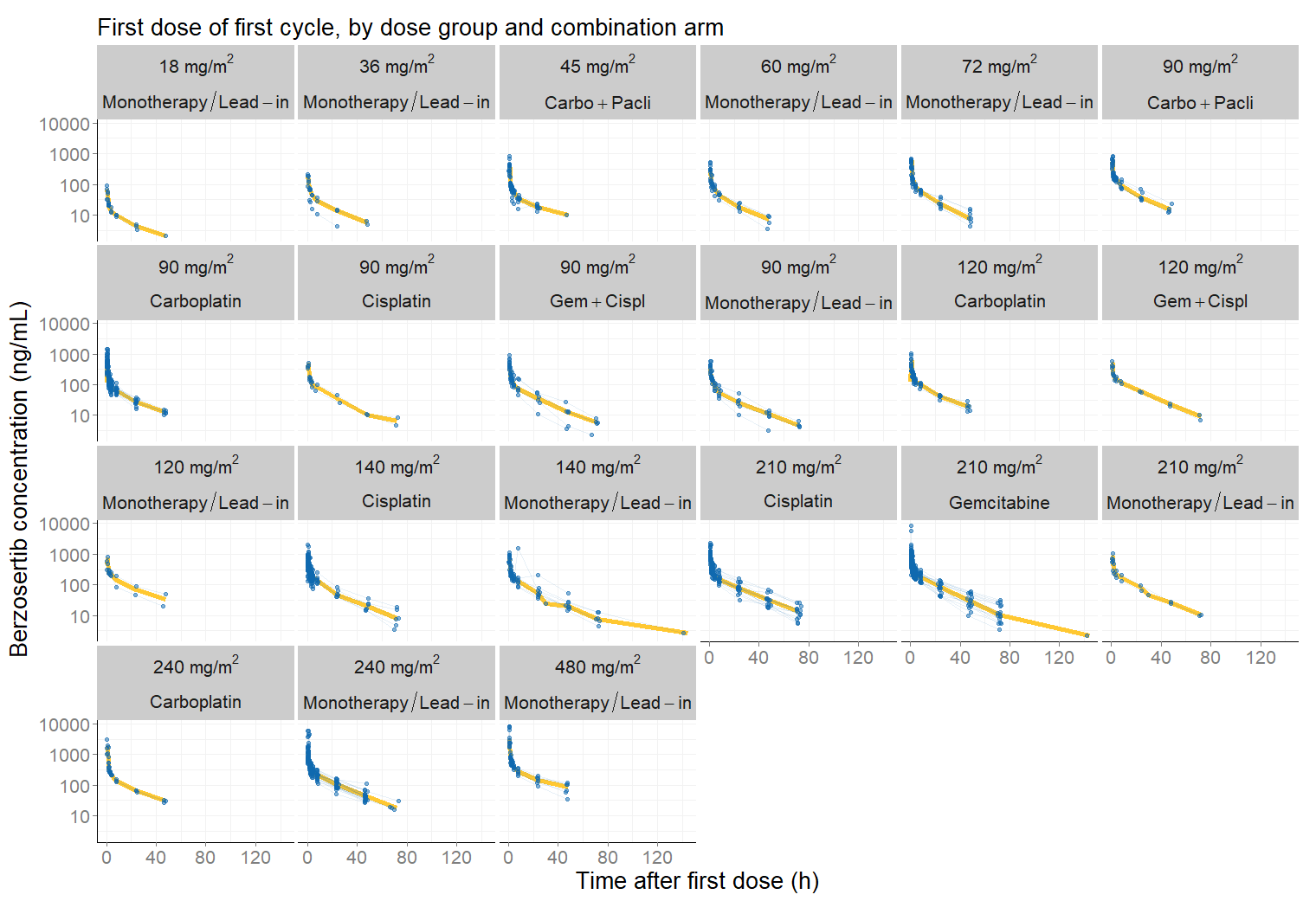


Carbo carboplatin, Cispl cisplatin, Gem gemcitabine, Pacli paclitaxel

Observed berzosertib concentrations are shown over time for each dose level and combination arm. Blue points connected by lines are individual observations; the yellow line a smoothing curve

**Fig. S3** Goodness-of-fit plots for the full covariate model
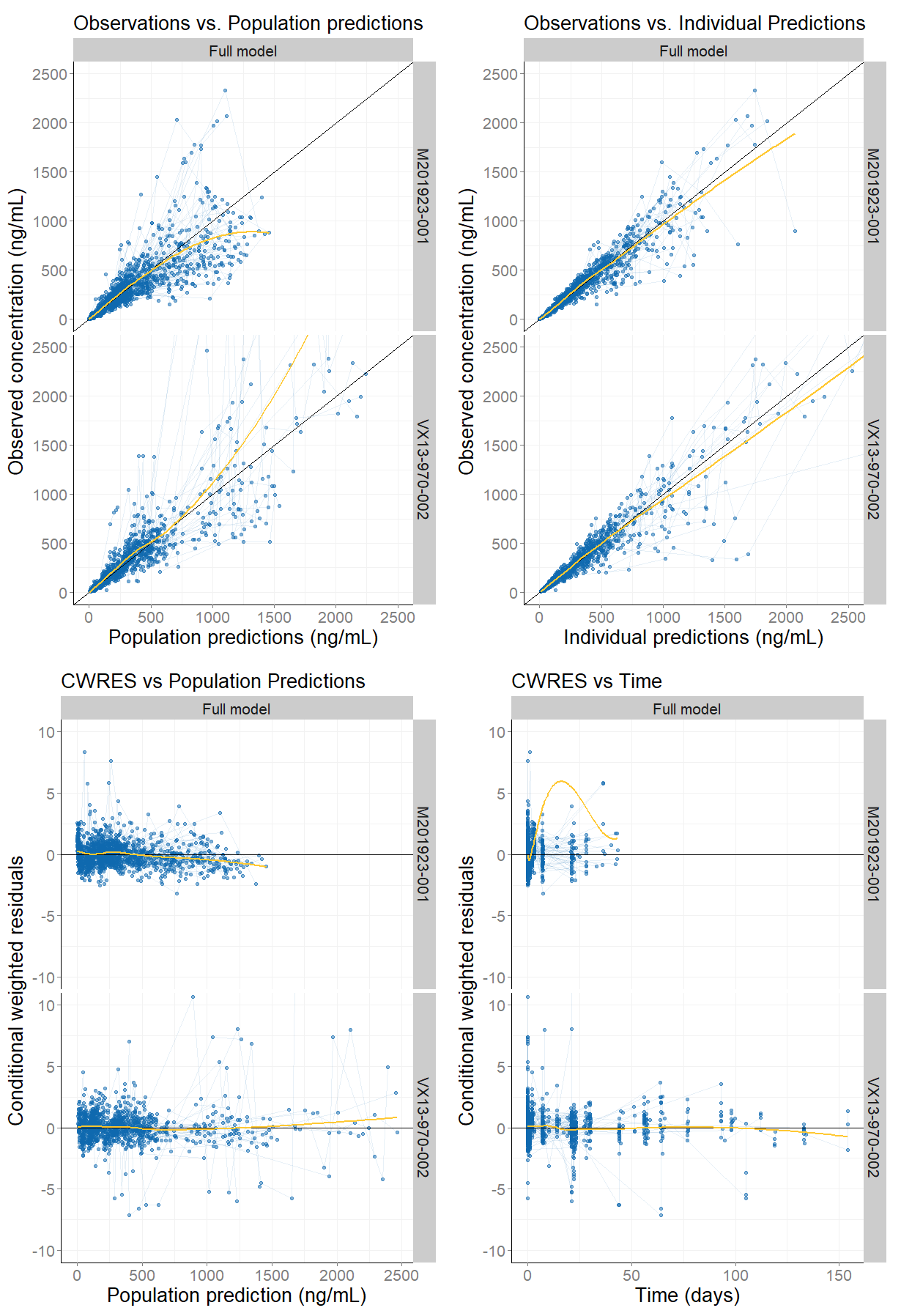


CWRES conditional weighted residuals

Goodness of fit plots of the full covariate model are shown

**Fig. S4** Visual Predictive Check for the full covariate model
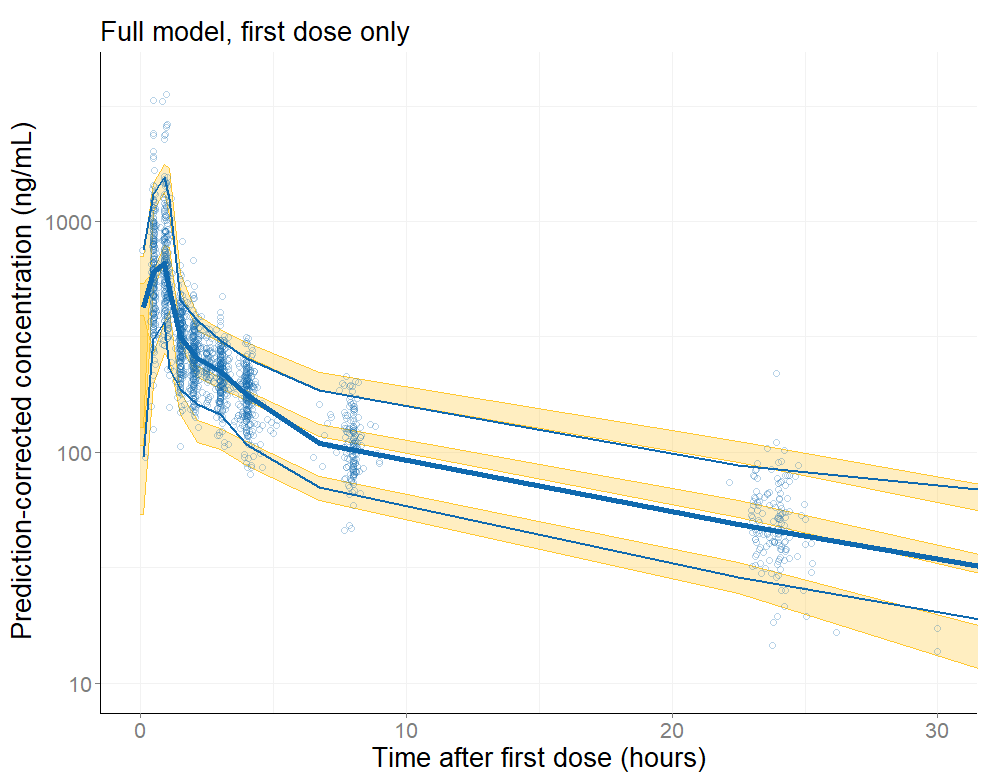


pcVPC prediction-corrected Visual Predictive Check

The pcVPC of the full covariate model is shown. Yellow shaded areas of the VPC represent 95% prediction intervals around the median and the 5^th^ and 95^th^ percentiles based on simulations (500 simulated datasets) following the first dose. Blue solid lines represent medians, 5^th^ and 95^th^ percentiles of the binned observed data. Points are individual observations

**Fig. S5** Representative individual predicted concentration time profiles from the two phase I studies


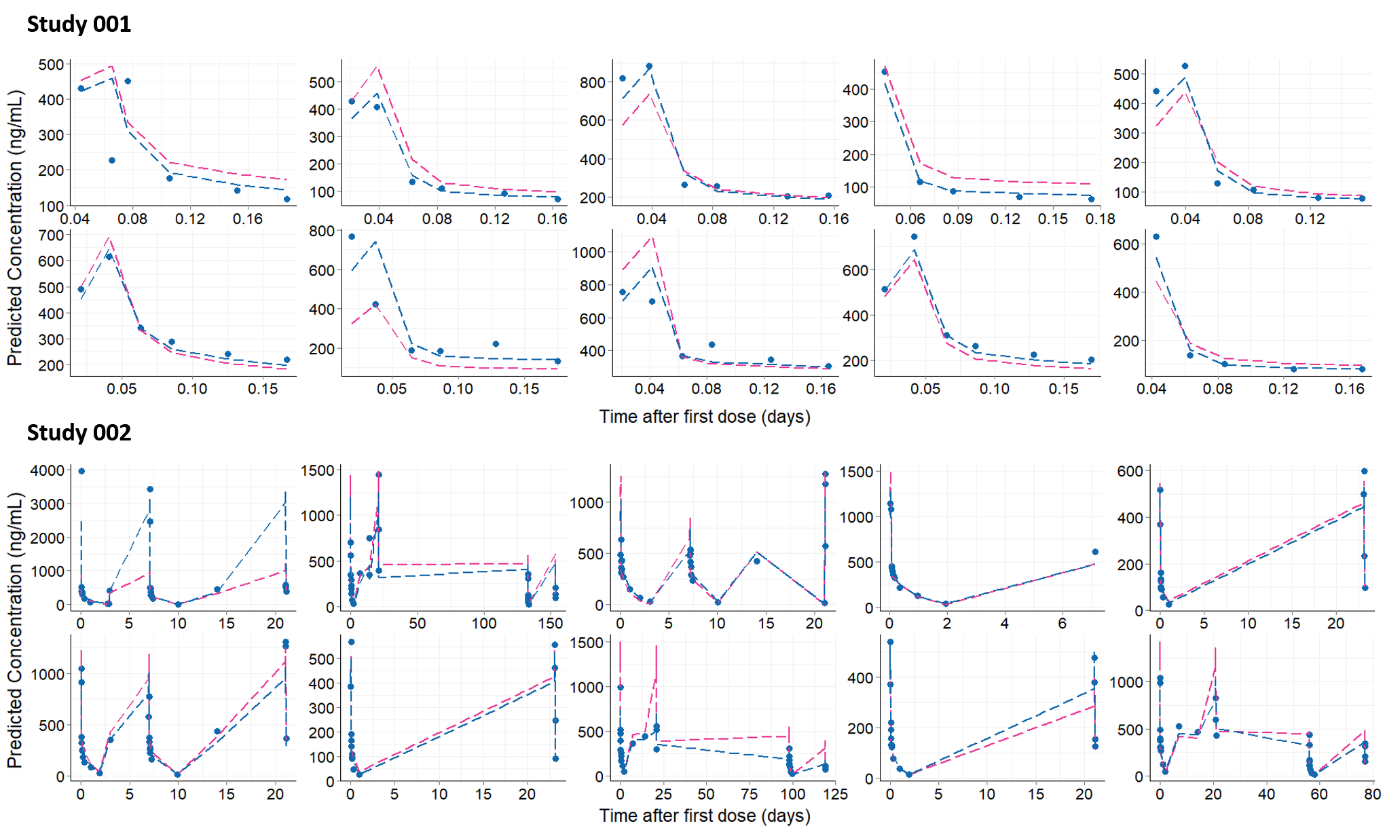
Representative individual predicted concentration time profiles overlaid with the observed concentrations from the two phase I studies. Pink and blue lines represent population and individual predictions, respectively. Points are individual observations

**Table S1** Demographics and baseline characteristics by study

| Variable | *Study MS201923-0001*  Value (*N* = 170) | *Study VX13-970-002*  Value (*N* = 70) |
| --- | --- | --- |
| *Demographics* |  |  |
| Age [years] | 59 {57.6} (26-79) [0] | 61.5 {60.4} (38-77) [0] |
| Bodyweight [kg] | 73 {75.2} (46-135) [0] | 72.3 {76} (48.2-150) [0] |
| BSA [kg/m^2^] | 1.82 {1.84} (1.4-2.52) [0] | 1.82 {1.84} (1.43-2.59) [0] |
| Lean body mass [kg] | 49.5 {52.4} (35.7-78.1) [1] | 50 {52} (36.9-78.9) [0] |
| Height [cm] | 168 {167} (99-191) [0] | 167 {167} (145-187) [0] |
| Sex [*n* (%)]  Male  Female | 68 (40%)  102 (60%) | 27 (38.6%)  43 (61.4%) |
| Race [*n* (%)]  White  Black  Asian  Other  Missing | 157 (92.4%)  3 (1.76%)  4 (2.35%)  4 (2.35%)  2 (1.18%) | 67 (95.7%)  0 (0%)  1 (1.43%)  2 (2.86%)  0 (0%) |
| Ethnicity [*n* (%)]  Hispanic/Latino  Not Hispanic/Latino  Missing | 7 (4.12%)  157 (92.4%)  6 (3.53%) | 0 (0%)  69 (98.6%)  1 (1.43%) |
| ***Lab values*** |  |  |
| Creatinine [µmol/L] | 66 {69.3} (32.7-148) [0] | 71 {71.2} (36-124) [0] |
| Creatinine clearance [mL/min] | 97.2 {102} (50-150) [0] | 89.2 {96.1} (57.2-150) [0] |
| Platelet count [10^9^ /L] | 264 {285} (93-816) [0] | 297 {298} (162-671) [0] |
| White cell count [cells /µL] | 7.2 {7.82} (1.37-25.2) [0] | 7.2 {7.24} (3.2-14.3) [0] |
| Albumin [g/L] | 39 {38.5} (23-49) [0] | 35.5 {35.4} (23-44) [0] |
| Bilirubin [µmol/L] | 6.84 {7.47} (0.5-20.5) [0] | 9 {9.84} (2-21) [0] |
| ALT [ U/L] | 19 {27.2} (4-178) [0] | 20.5 {26.3} (8-159) [0] |
| AST [U/L] | 24 {31.2} (10-159) [0] | 26 {30.8} (9-115) [0] |
| Renal impairment [*n* (%)]  None  Mild  Moderate  Severe | 104 (61.2%)  52 (30.6%)  14 (8.24%)  0 (0%) | 33 (47.1%)  36 (51.4%)  1 (1.43%)  0 (0%) |
| Hepatic impairment [*n* (%)]  None  Mild  Moderate  Severe  Missing | 109 (64.1%)  30 (17.6%)  0 (0%)  1 (0.588%)  30 (17.6%) | 57 (81.4%)  13 (18.6%)  0 (0%)  0 (0%)  0 (0%) |
| ***Disease status*** |  |  |
| Tumor burden [mm] | 80.5 {91.5} (10-312) [0] | 56 {66} (12-169) [0] |
| Tumor type [n (%)]  NSCLC  TNBC  SCLC  PrCa  Breast  H&N  CRC  Ovarian  Mesothelioma  Other | 46 (27.1%)  33 (19.4%)  14 (8.24%)  5 (2.94%)  6 (3.53%)  1 (0.588%)  26 (15.3%)  4 (2.35%)  8 (4.71%)  27 (15.9%) | 2 (2.86%)  0 (0%)  2 (2.86%)  2 (2.86%)  5 (7.14%)  0 (0%)  18 (25.7%)  5 (7.14%)  4 (5.71%)  32 (45.7%) |
| ECOG PS [*n* (%)]  0  1  2  3 | 46 (27.1%)  113 (66.5%)  5 (2.94%)  6 (3.53%) | 19 (27.1%)  51 (72.9%)  0 (0%)  0 (0%) |

*ALT* alanine transaminase, *AST* aspartate transaminase, *BSA* body surface area, *CRC* colorectal cancer, *ECOG* *PS* Eastern Cooperative Oncology Group Performance Status *H&N* head & neck, *NSCLC* non-small cell lung cancer, *PrCa* prostate cancer, *SCLC* small cell lung cancer, *TNBC* triple negative breast cancer,

Continuous covariates are reported as median {geometric mean} (range) [missing]

**Table S2** Population parameter estimates for the base model

| Parameter | Estimate | RSE%^a^ | Asymptotic 95% CI | Shrinkage (%)^b^ |
| --- | --- | --- | --- | --- |
| Clearance CL [L/h] | 56 | 2.5 | 54-59 | - |
| Central volume V1 [L] | 111 | 4.4 | 100-120 | - |
| Peripheral volume V2 [L] | 861 | 2.1 | 830-900 | - |
| Intercompartmental clearance Q [L/h] | 296 | 3.3 | 280-320 | - |
| IIV on CL [var] | 0.081 | 6.2 | 0.062-0.1 | 8.6 |
| cov (CL, V1) | 0.067 | 13.0 | 0.032-0.1 | - |
| cov (CL, Q) | 0.048 | 14.0 | 0.021-0.075 | - |
| cov (CL, V2) | 0.057 | 6.5 | 0.042-0.071 | - |
| IIV on V1 [var] | 0.38 | 7.1 | 0.28-0.49 | 8 |
| cov (V1, Q) | 0.26 | 8.3 | 0.18-0.35 | - |
| cov (V1, V2) | 0.093 | 9.5 | 0.058-0.13 | - |
| IIV on Q [var] | 0.24 | 8.9 | 0.15-0.32 | 5.8 |
| cov (Q, V2) | 0.089 | 14.0 | 0.021-0.075 | - |
| IIV on Q [var] | 0.071 | 6.5 | 0.053-0.089 | 6.4 |
| Proportional residual error [sd] | 0.22 | 4.1 | 0.21-0.24 | - |
| Additive residual error [ng/mL] | 1.9 | 13.0 | 1.4-2.4 | - |

*CI* confidence interval, *CL* clearance, *var* variance, *cov* covariance, *sd* standard deviation, *IIV* inter-individual variability, *Q* intercompartmental clearance, *RSE* relative standard error, *V1* central volume of distribution, *V2* peripheral volume of distribution

^a^Obtained by NONMEM covariance step. The relative standard errors for individual variability parameters are reported on the approximate standard deviation scale (standard error/variance estimate)/2

^b^The epsilon shrinkage was estimated to be 11%
